# Cardiac Oscillations Complicating Brain Death Diagnosis

**DOI:** 10.1155/2023/1132406

**Published:** 2023-09-11

**Authors:** Brittany Bolt, Farid Muakkassa, Lindsay Bruening, Cameron Marcus, Brittany Cunningham, Erin Pawlak, Richard Gandee, Christopher Newey

**Affiliations:** ^1^Cerebrovascular Center, Neurological Institute, Cleveland Clinic Foundation, Cleveland, Ohio, USA; ^2^Section of Trauma, Cleveland Clinic Akron General Hospital, Akron, Ohio, USA; ^3^Section of Neurology, Cleveland Clinic Akron General Hospital, Akron, Ohio, USA; ^4^Section of Emergency Medicine, Cleveland Clinic Akron General Hospital, Akron, Ohio, USA; ^5^Section of Pharmacy, Cleveland Clinic Akron General Hospital, Akron, Ohio, USA; ^6^Department of Neurocritical Care and ICU-EEG, Sanford Health, Sioux Falls, OH, USA

## Abstract

Death by neurologic criteria (DNC) or brain death is a clinical diagnosis. It is often complicated by variations in policies as well as confounders on examination. We discuss here the case of a 27-year-old male who had a cardiac arrest following toxic gaseous exposure. He ultimately progressed to brain death but was identified as having cardiac oscillations during clinical assessments that complicated the diagnosis. We discuss the case as well as the maneuvers used to clarify that the “triggered breaths” on the ventilator were indeed cardiac oscillations.

## 1. Introduction

Death by neurologic criteria (DNC; also referred to as brain death) is an important and sensitive process that requires a great degree of care and precision [1]. DNC is a clinical diagnosis [2]. Although there are established criteria widely used to declare brain death, the examination is not always straightforward as there can be multiple confounding factors interfering with the examination [2, 3]. Criteria for DNC require identifying an irreversible and proximate injury causing coma followed by addressing confounders, performing a neurologic examination including apnea testing, and documenting the findings [2]. Despite this simplicity, variations to hospital policies exist [4, 5]. In addition to the confusion surrounding policies, brain death-associated movements can confound the diagnosis [3]. Some of these include extreme findings such as the Lazarus sign and head turning phenomena, as well as the less extreme such as undulating toe or finger flexion to noxious stimulation [3, 6]. Lazarus sign, where the patient exhibits arm flexion along with neck flexion, and head turning, where there may be extension of the arms with head turning to stimulation, can lead to emotional distress to the bedside providers and families ultimately resulting in delayed brain death declaration [3, 6, 7]. However, it is crucial for the clinician to recognize that confounders can exist and must distinguish them from cerebral mediated findings.

In addition to brain death-associated movements, other confounders exist. One such confounder that is difficult to distinguish is cardiac oscillations. Cardiac oscillations refer to the waves produced by cardiac activity, which can impact ventilator response especially with a sensitive flow triggering [8–10]. The degree of cardiac oscillations varies, typically between 0 and 2 cm H_2_O [8–10]. These oscillations result in the ventilator “recognizing” patient effort, which leads to delivering a supported breath. This can falsely give the impression that a patient is taking spontaneous breaths when they are not. The cause for cardiac oscillations is typically the flow trigger sensitivity on the ventilator [8–10].

We report a case of brain death declaration where cardiac oscillations interfered with examination leading to difficulties in DNC declaration. We also discuss the technique used to ultimately determine cardiac oscillations.

## 2. Case

**Figure 1:**
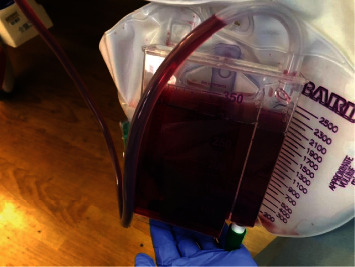
Wine-colored urine. Image shows typical finding in patients who receive hydroxocobalamin as treatment following a toxic exposure. Our patient received hydroxocobalamin following exposure to hydrogen sulfide gas poisoning.

**Figure 2:**
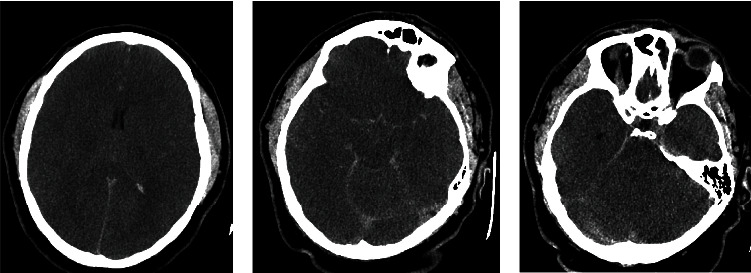
Computed tomography (CT) of the head (CTH). CTH showing diffuse cerebral edema (a) and pseudo-subarachnoid hemorrhage pattern (b, c).

**Figure 3:**
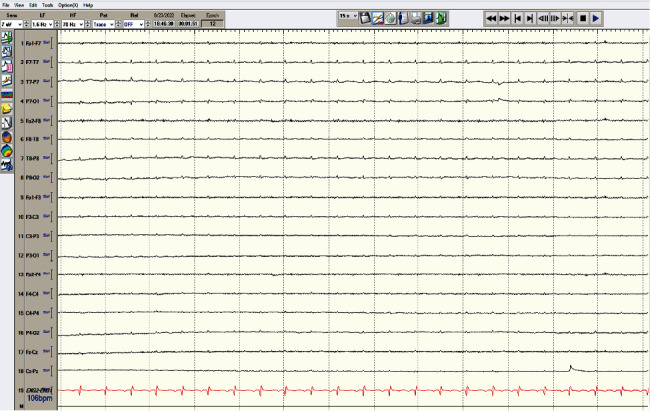
Electroencephalography (EEG): EEG shows electrocerebral inactivity with diffuse cardiogenic artifact. Bipolar montage, sensitivity 7 *μ*v, LF 1.6 Hz, and HF 70 Hz.

**Figure 4:**
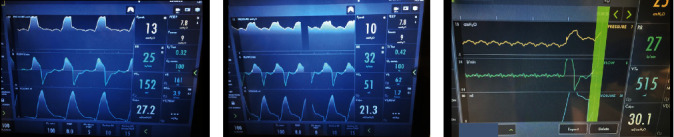
Mechanical ventilator waveforms:(a) pressure support (PS) ventilation trial using 100% FiO_2_, positive end-expiratory pressure (PEEP) of 5 cmH_2_O, and pressure support above PEEP of 5 showing respiratory rate of 25 breaths/min and tidal volume of 152 mL; (b) changing ventilator to a continuous positive airway pressure (CPAP) trial of 100% FiO_2_, PEEP of 5 cmH_2_O, and pressure support above PEEP of 0 shows respiratory rate of 32 breaths/min and tidal volume of 51 mL. (a) and (b) were with a flow trigger setting at 4 liters per minute; (c) with changing the flow trigger to a pressure trigger (-2 cmH_2_O), the pressure and flow waveforms showed a cardiac oscillation pattern which was independent from the heart frequency or heart rate which was 96-102. The increase in peak pressure and flow volume seen at the end of the recording was during an expiratory hold maneuver.

**Figure 5:**
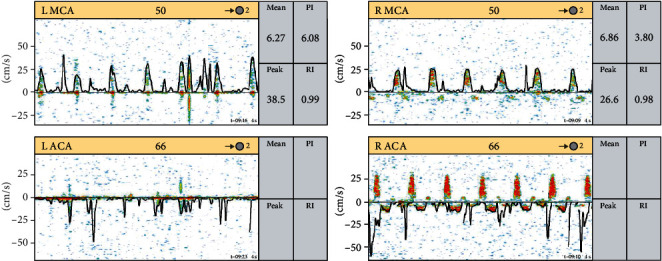
Transcranial Doppler (TCD): TCD showing typical systolic spikes in the middle cerebral artery (MCA) bilaterally and the “to-fro” pattern in the anterior cerebral artery (ACA) territory best seen on the right.

A 27-year-old male presented in cardiac arrest to an outside facility following gaseous exposure to hydrogen sulfide. He was found slumped over in an excavator at his employment at a toxic waste facility. In the field, he was in pulseless electrical activity (PEA). Advanced cardiac life support (ACLS) was started. Return of spontaneous circulation occurred after three rounds of ACLS. At the outside hospital, he was decontaminated and treated with hydroxocobalamin, which resulted in bodily fluids turning a wine color (Figure 1). He was transferred to a tertiary care center. On arrival, approximately 1 hour following cardiac arrest, neurological examination was poor. He had nonreactive, mid-sized pupils, no corneal reflex, no oculocephalic reflex, no oculovestibular reflex, no cough, and no gag. Computed tomography (CT) of the head was obtained (Figure 2). The CT of the head showed diffuse cerebral edema and pseudo-subarachnoid hemorrhage pattern (Figures 2(a)–2(c)). Over the next few hours, he developed increased urine output of 900 mL and increasing sodium consistent with diabetes insipidus. He was treated with 2 mcg IV of desmopressin acetate (DDAVP) which slowed the urine output. Electroencephalography (EEG) showed electrocerebral inactivity (<2 *μ*v) with diffuse cardiogenic artifact (Figure 3). He was noted to have “spontaneous breathing” on the ventilator despite adjustments to the flow sensitivity (Figures 4(a) and 4(b)). When disconnected briefly from the ventilator and observed, he was apneic which confirmed that the ventilator was likely being triggered by cardiac oscillations. Apnea testing occurred after preoxygenating. PaCO_2_ at the start of testing was 38 mmHg. He was disconnected from the ventilator. 6 L of O_2_ was provided via catheter in the endotracheal tube. After over 8 minutes of apnea, his PaCO_2_ increased to 71 mmHg. Given the ongoing concern for the ventilator findings (Figures 4(a) and 4(b)), the ventilator was replaced with an alternate and ancillary testing was recommended. The alternate ventilator allowed for adjustment of the trigger settings from a flow trigger to a pressure trigger setting, enabling cardiac oscillations to be easily visualized on the ventilator (Figure 4(c)). Transcranial Doppler (TCD) was obtained and showed systolic spikes in the middle cerebral artery (MCA) bilaterally with the “to-fro” pattern in the anterior cerebral artery (ACA) territory best seen on the right (Figure 5). Following testing and discussions, death by neurologic criteria was confirmed.

## 3. Discussion

This case highlights the importance of recognizing ventilator autotriggering in patients who otherwise meet death by neurologic criteria. In this case, cardiac oscillations were the etiology for ventilator autotriggering.

Ventilator autotriggering is defined as the triggering of the ventilator in the absence of patient effort, intrinsic respiratory drive, or inspiratory muscle activity [8]. Ventilator autotriggering can be either extrinsic or intrinsic. The common extrinsic causes are excessive condensation, humidification, endotracheal tube cuff inflation or leak, leaks in other ventilator circuit connections, malposition of the inline suction catheter, chest tube leaks, or noise within the ventilator circuit due to movement or vibration [11]. The common intrinsic cause is cariogenic respiratory oscillation [11]. The pathophysiology for cardiogenic respiratory oscillation is based on the cyclic cardiac stroke volume and displacement of compliant air-filled lung tissue ultimately causing gas movement [11]. During systole, the heart ejects blood causing the intrathoracic volume to decrease [11]. This causes a negative intrathoracic pressure [11]. With the cyclic variations of pulmonary blood flow from the cardiac cycles, there is a shift in pulmonary gas volume altering the airway pressure and flow [11]. This results in a ventilator autotrigger. This is often seen in patients with a hyperdynamic precordium [10, 11]. While cariogenic respiratory oscillation may initiate autotriggering, extrinsic factors as listed above may potentiate persistent ongoing oscillations that lead to autotriggering. We highlight the EEG in this patient due to the robust, diffuse cardiogenic artifact seen.

Ventilator autotriggering is often caused by improper setting of trigger threshold [11]. The two main types of ventilator triggers are flow trigger and pressure trigger. The flow trigger mode uses a continuous base flow setting and flow threshold trigger setting [11]. The sensitivity setting is typically around 4 liters per minute. The ventilator will initiate a breath or register a breath if the flow measured at the exhalation valve drops below the set sensitivity [11]. In contrast, the pressure trigger mode requires the ventilator to maintain a set pressure. Once the pressure drops below the set pressure threshold (typically -2 cmH_2_O), the ventilator will register a breath [11]. The flow trigger is more sensitive and thus prone for false triggering by cardiac oscillations. Often, the bedside caregiver will increase the flow sensitivity to create a need for a higher inspiratory effort to deliver a breath. If this does not remedy the autotriggering, changing to pressure trigger mode is recommended. Ultimately, disconnecting the patient from the ventilator and observing respiratory effort is the best practice to determine if the inspirations are cardiac oscillations or not as was employed in our patient's case [11–15]. Once disconnected, there was no respiratory effort, which clarified the initial confusion.

The American Academy of Neurology guidelines on determination of brain death [2] discuss “absence of a breathing drive” using a “CO_2_ challenge” immediately after the absence of other brainstem reflexes. There is no discussion of ensuring no respiratory effort prior to apnea such as placing patient on a CPAP trial. This is often misleading in many institutional policies. An evaluation of 508 unique hospital policies on brain death determination found that 305 (62.1%) required documentation of absent spontaneous respirations on mechanical ventilation [4]. This requirement in documentation can lead to challenges particularly with ventilator autotriggering.

The lack of recognizing ventilator autotriggering can delay the pronouncement of a brain dead patient. This can cause unnecessary, additional emotional distress on the ICU team and family [11]. The brain dead patient can become unstable. Lustbader et al. showed that delaying diagnosing brain death increases the likelihood of a cardiac arrest [16, 17]. Also, prolonged exposure to the proinflammatory state that occurs after brain death can adversely affect allografts and graft survivals [18]. Our patient did not have any adverse effects on donation viability due to the brief delay in donation. We provided support to the bedside caregivers with additional testing to support the clinical diagnosis of brain death. We did obtain the TCDs which supported the diagnosis. Illustrating the lack of cerebral blood flow was reassuring to those who cared for the patient.

This case highlights the importance of recognizing confounders that may occur during the diagnosis of brain death. In our case, the confounder was the cardiac oscillations. We highlight what we did to illustrate that the presumed respiratory efforts were indeed cardiac respiratory effort. Transiently disconnecting the patient from the ventilator and observing for respiratory effort clarified the cardiac oscillation confounder. We also highlight the additional steps we took to provide data to the bedside caregivers to support them emotionally.

## Data Availability

Data is readily available to the authors in the hospital electronic medical record.
